# Is There an Association between Concurrent Epstein–Barr Virus Infection and Sudden Hearing Loss?—A Case—Control Study in an East Asian Population

**DOI:** 10.3390/jcm12051946

**Published:** 2023-03-01

**Authors:** Wei-Lun Lan, Chih-Hao Chen, Yuan-Chia Chu, Yen-Fu Cheng, Chii-Yuan Huang

**Affiliations:** 1 Department of Otolaryngology-Head and Neck Surgery, Taipei Veterans General Hospital, Taipei 112, Taiwan; 2 Information Management Office, Taipei Veterans General Hospital, Taipei 112, Taiwan; 3 Medical AI Development Center, Taipei Veterans General Hospital, Taipei 112, Taiwan; 4 Department of Information Management, National Taipei University of Nursing and Health, Taipei 112, Taiwan; 5 School of Medicine, National Yang Ming Chiao Tung University, Taipei 112, Taiwan; 6 Department of Medical Research, Taipei Veterans General Hospital, Taipei 112, Taiwan; 7 Institute of Brain Science, National Yang Ming Chiao Tung University, Taipei 112, Taiwan

**Keywords:** sudden sensorineural hearing loss, Epstein–Barr virus, case–control study

## Abstract

Viral infection serves as the crucial etiology for the development of sudden sensorineural hearing loss (SSNHL). We aimed to investigate whether there is an association between concurrent Epstein–Barr virus (EBV) infection and SSNHL in an East Asian population. Patients who were older than 18 years of age and met the criteria of sudden hearing loss without an identifiable etiology were enrolled from July 2021 until June 2022, followed by the serological testing of IgA antibody responses against EBV-specific early antigen (EA) and viral capsid antigen (VCA) with an indirect hemagglutination assay (IHA) and real-time quantitative polymerase chain reaction (qPCR) of EBV DNA in serum before the treatment was initiated. After the treatment for SSNHL, post-treatment audiometry was performed to record the treatment response and degree of recovery. Among the 29 patients included during enrollment, 3 (10.3%) had a positive qPCR result for EBV. In addition, a trend of poor recovery of hearing thresholds was noted for those patients with a higher viral PCR titer. This is the first study to use real-time PCR to detect possible concurrent EBV infection in SSNHL. Our study demonstrated that approximately one-tenth of the enrolled SSNHL patients had evidence of concurrent EBV infection, as reflected by the positive qPCR test results, and a negative trend between hearing gain and the viral DNA PCR level was found within the affected cohort after steroid therapy. These findings indicate a possible role for EBV infection in East Asian patients with SSNHL. Further larger-scale research is needed to better understand the potential role and underlying mechanism of viral infection in the etiology of SSNHL.

## 1. Introduction

Sudden sensorineural hearing loss (SSNHL) is a common otologic emergency that refers to sudden-onset and unexplained hearing loss. SSNHL is defined as hearing loss at a decibel hearing level (dB HL) of 30 or greater in three consecutive frequencies that develops within 3 days or less [1]. Previous evidence has shown that the incidence of sudden sensorineural hearing loss ranges from 5 to 160 cases per 100,000 persons per year [2,3,4,5,6]. Although this disease is not highly prevalent, sudden hearing loss serves as a common etiologic condition that can cause disability in hearing and long-term sequelae, including anxiety and depression, given its sudden onset and management [7,8].

Currently, the pathogenesis of SSNHL has not been well elucidated. It is often considered to be related to infectious, autoimmune, and blood circulation disorders [5,9,10]. Given these potential underlying etiologies, glucocorticoids along with antioxidant and vessel dilatation agents, which improve the microcirculation of the inner ear, promote the supply of oxygen, and improve immunity, remain the mainstream management strategy for SSNHL [11,12,13]. Despite research efforts toward the design of pharmacological strategies for the disease, inconsistencies and unpredictability in recovery after treatment are still found. Additionally, the role of a viral etiology in the origin of the disease has been considered since the disease was first addressed [14]. To date, the causative agents for SSNHL include mumps virus, the well-known virus that accounts for 7% of all hearing loss from viral infections, followed by the herpes zoster (i.e., herpes zoster oticus) and rubella [15,16]. Although all the current studies remain small and have inadequate study designs, the viral etiology serves as a central part of the pathophysiology of sudden hearing loss [6,17].

The Epstein–Barr virus (EBV), also recognized as human herpesvirus 4, is a member of the herpesvirus family. It is one of the most common human viruses, disseminates most commonly via body fluids (e.g., saliva), is a frequent cause of infectious mononucleosis, and can even induce various malignancies (e.g., lymphoma, gastric cancer, etc.) [18,19,20]. In East Asia, including Taiwan, EBV represents a highly prevalent viral infection. According to a previous, large-scale epidemiological investigation in Taiwan, over 95% of the general population older than 18 is seropositive for EBV [21], which means a previous or ongoing EBV infection. The possible link between EBV infection and sudden hearing loss has been repeatedly addressed in previous case reports or series among the potential causative viruses related to sudden hearing loss [22,23,24]. Despite the theoretical role of EBV in SSNHL, direct evidence regarding the association between EBV and SSNHL is insufficient and remains controversial. Thus, we aimed to perform a case–control study to investigate whether there is an association between concurrent EBV infection and SSNHL.

## 2. Methods

### 2.1. Subject Enrollment

This case–control study was conducted from July 2021 to June 2022, and patients were included when the following criteria were met: Patients were older than 18 years of age and had sudden hearing loss without an identifiable etiology. Patients who were younger than 18 years of age or patients with hearing loss from an identified etiology of sensorineural hearing loss (e.g., otitis media, drug-induced, noise-induced, Meniere’s disease, herpes zoster oticus, or acoustic neuroma) or genetic deafness (e.g., GJB2 mutation) were regarded as ineligible. The study was performed with informed consent from all the participants and under the approval of the Institutional Review Board of Taipei Veterans General Hospital (IRB number: 2020-06-001AC).

### 2.2. Audiological Examination

All the patients underwent pure-tone audiometry (PTA) for the diagnosis of sudden hearing loss at presentation and the post-treatment follow-up. The eligible patients also underwent tympanometry, the auditory brainstem evoked response test, and serum examination to rule out identifiable otologic causes (e.g., conduction abnormality, acoustic neuroma, and otitis media). The average PTA was calculated for the thresholds of 0.5, 1, 2, and 4 kHz. Audiograms were obtained at the initial visit (pretreatment) and at 1 week, 2 weeks, 1 month, and 2 months after treatment. The grade of the initial audiogram was recorded using the modified Siegel criteria proposed by our previous study [25]. According to these criteria, hearing severity is classified into five grades: Grade 1, a mean hearing level of ≤25 dB HL; Grade 2, a mean hearing level of 26–45 dB HL; Grade 3, a mean hearing level of 46–75 dB HL; Grade 4, a mean hearing level of 76–90 dB HL; and Grade 5, a mean hearing level of >90 dB HL. The last measured average thresholds were defined as the final hearing levels. Additionally, the audiogram shape was recorded and categorized into ascending, descending, flat, and profound, as defined by Qian et al. [26]. The ascending type included cases whose average hearing threshold at 0.25 to 0.5 kHz was 20 dB higher than that at 4 to 8 kHz. The descending type included cases where the average hearing threshold at 4 to 8 kHz was 20 dB higher than that at 0.25 to 0.5 kHz. The flat type referred to cases whose threshold was observed to be similar across the entire frequency range, and the hearing threshold did not exceed 80 dB HL. For patients with a flat audiogram and a hearing threshold over 80 dB, the audiogram shape was classified as profound.

### 2.3. Serological Examination

DNA and antibodies from the circulating Epstein–Barr virus (EBV) represent two of the most sensitive peripheral blood markers of EBV infection [27]. All the eligible cases underwent a serological examination before treatment was initiated. The samples were stored at −80 degrees Celsius until analysis. IgA antibodies against EBV-specific early antigen (EA) and viral capsid antigen (VCA) were tested with commercial kits (EUROIMMUN, Lübeck, Germany) using indirect immunofluorescence assays (IFAs). Following the manufacturer’s instructions, plasma samples were screened at a dilution of 1:40, followed by twofold serial dilutions. An EBV IgA titer less than 1:40 was undetectable. DNA from circulating EBV was tested using the real-time quantitative polymerase chain reaction (qPCR) method (Cobas 6800 System EBV Test, Roche, Basel, Switzerland), with a detection limit of 35 IU per milliliter (IU/mL) (1 IU/mL = 0.62 copies/mL). The parameters for PCR amplification and cycling were based on the recommendations of the manufacturer.

### 2.4. Management of SSNHL and Follow Up

All the patients received the same standard treatment of intravenous steroids (i.e., dexamethasone at 5 mg twice daily for five days) and oral steroids (i.e., prednisolone at 1 mg/kg, tapered within the following five days), followed by post-treatment audiometry to record the treatment response and degree of recovery. The degree of hearing recovery was evaluated based on the modified Siegel criteria and classified into one of five groups as follows: complete recovery (CR), partial recovery (PR), slight improvement (SI), no improvement (NI), and nonserviceable ear (NS) [25]. CR was defined as a final hearing threshold better than 25 dB HL. PR was defined as a hearing gain greater than 15 dB HL and a final hearing threshold of 26–45 dB HL. SI was defined as a hearing gain of more than 15 dB HL and a final hearing threshold of 46–75 dB HL. NI meant a hearing gain of less than 15 dB HL or a final hearing threshold of 76–90 dB HL. NS was defined as a final hearing threshold of less than 90 dB HL [25]. Analysis and comparison of features between patients with negative PCR results and positive PCR results were executed according to the protocol.

### 2.5. Statistical Analysis

Comparisons of continuous variables between the two groups were performed with Mann—Whitney U tests as indicated, while comparisons of dichotomous data within groups were conducted using Fisher’s exact tests as indicated. All statistical tests were two-sided, and the level of significance was set at 5%. A *p*-value less than 0.05 was considered significant. All statistical calculations were performed with SPSS version 20.

## 3. Results

### 3.1. Baseline Characteristics

A total of 29 patients were included during enrollment. All of them presented with unilateral SSNHL. The mean age of the entire cohort was 46.0 ± 14.7 years, with 14 females and 15 males. Of those, 3 patients received a positive EBV PCR result, while the other 26 patients tested negative for EBV using PCR. Among those with negative PCR results, 12 patients (42.6%) were females, and 14 patients (53.8%) were males, while there were 2 females (66.7%) and 1 male (33.3%) with positive PCR results. Furthermore, 15 patients (57.7%) with undetectable IgA titers and 11 patients (42.3%) with detectable IgA titers were found in the PCR-negative group, while 2 patients (66.7%) with undetectable titers and 1 patient (33.3%) with detectable titers were found in the PCR-positive group. There were no differences in the detectable IgA ratio between the PCR-positive and PCR-negative groups (*p* = 1.00). VCA-IgA and EA-IgA titer distributions are listed (Table 1). Twelve patients (41.4%) had detectable EBV-specific VCA IgA (≥1:40), including nine patients who exhibited a titer of 1:40, two patients who had a titer of 1:80, and one patient who had a titer of 1:160. On the other hand, one patient (3.4%) exhibited elevated IgA titers of 1:80 and 1:40 against both an EBV-specific EA IgA and a VCA IgA, respectively. Regarding the audiometric curve shape, both PCR-negative and PCR-positive patients predominantly demonstrated profound (42.3% vs. 66.7%) and flat-type audiometric curves (34.6% vs. 33.3%). Among the PCR-negative group, one patient (3.8%) had Grade 1 hearing loss, four patients (15.4%) had Grade 2 hearing loss, eight patients (30.8%) had Grade 3 hearing loss, four patients (15.4%) had Grade 4 hearing loss, and nine patients (34.6%) had Grade 5 hearing loss. Among the positive PCR group, one patient (33.3%) had Grade 3 hearing loss, and two patients (66.7%) had Grade 4 hearing loss. Comparisons between PCR-negative and PCR-positive groups based on age, sex, detectable IgA antibody or not, and pretreatment hearing thresholds did not demonstrate a significant difference (Table 1).

### 3.2. Hearing Recovery

During the follow-up, 2 of the eligible patients dropped out, and thus a total of 27 patients were included in the outcome evaluation. Among the 24 patients with negative PCR results, CR was observed in 8 patients (33.3%), PR was observed in 2 patients (8.3%), SI was observed in 3 patients (12.5%), NI was observed in 8 patients (33.3%), and NS was found in 3 patients (33.3%). In addition, the results of the three patients with positive PCR showed one patient with CR, one patient with PR, and one patient with NI. The average hearing gain, compared with the initial presentation, was 25.7 dB HL and 33.3 dB HL in the PCR-negative group and PCR-positive group, respectively, without a significant difference (*p* = 0.635) (Table 2).

### 3.3. Features of Patients with Positive PCR Results

**Figure 1:**
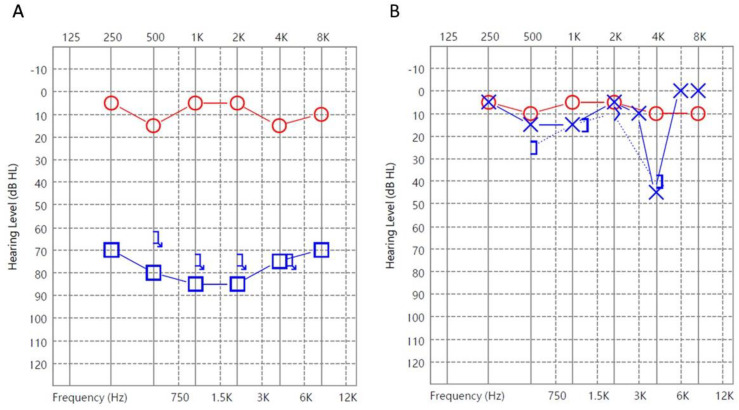
Audiograms of Patient 1 with positive PCR: (**A**) at presentation and (**B**) 2 months after treatment. (O: Unmasked air conduction thresholds in the right ear; X: Unmasked air conduction in the left ear; ]: Masked bone conduction in the left ear; ☐: Masked air conduction in the left ear; Arrows (on any symbol): No response).

Among the three patients with sudden hearing loss and positive PCR results, two (66.7%) had the profound type, and one (33.3%) had the flat type. The first patient was a 39-year-old female presenting with a hearing level of 80 dB HL. The serum level of EBV DNA in this patient was 35 IU/mL (21.7 copies/mL), and the patient showed complete recovery with a hearing gain of 61 dB (Figure 1).

**Figure 2:**
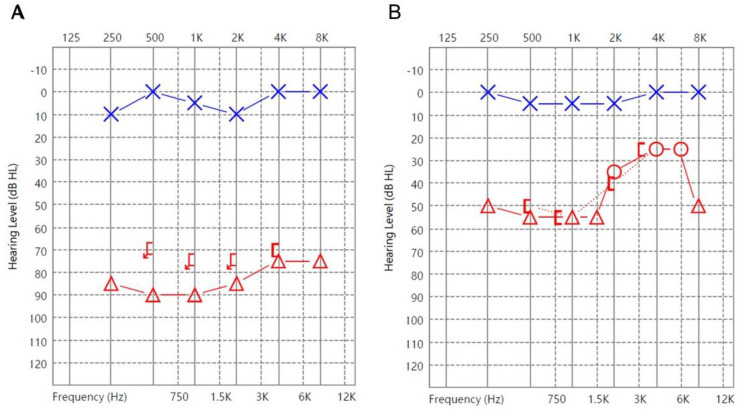
Audiograms of Patient 2 with positive PCR: (**A**) at presentation and (**B**) 2 months after treatment. (O: Unmasked air conduction thresholds in the right ear; X: Unmasked air conduction in the left ear; [: Masked bone conduction in the right ear; Δ: Masked air conduction in the right ear; Arrows (on any symbol): No response).

The second patient was a 26-year-old female presenting with a hearing level of 85 dB HL. While the serum level of EBV DNA in this patient was 59 IU/mL (36.58 copies/mL), the patient demonstrated good recovery with a hearing gain of 42 dB (Figure 2).

**Figure 3:**
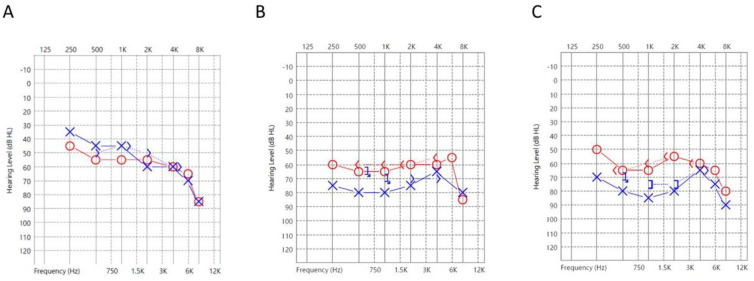
Audiograms of Patient 3 with positive PCR: (**A**) 6 weeks before presentation; (**B**) at presentation; (**C**) 2 months after treatment. (O: Unmasked air conduction thresholds in the right ear; X: Unmasked air conduction in the left ear; <: Unmasked bone conduction in the right ear; >: Unmasked bone conduction in the left ear; ]: Masked bone conduction in the left ear; Arrows (on any symbol): No response).

The last patient was a 46-year-old male with a left hearing level of 75 dB HL (Figure 3B). This patient had a history of tinnitus and bilateral sensorineural hearing loss. He received serial hearing tests for a tinnitus evaluation. A PTA taken six weeks before this episode showed bilateral sensorineural hearing loss (Figure 3A). The patient presented a sudden deterioration of the pre-existing hearing loss (Figure 3B), and left idiopathic SSNHL was diagnosed after a thorough evaluation to exclude relevant causes. The patient had the highest serum level of EBV DNA at 473 IU/mL (293.26 copies/mL), suggesting the most active EBV infection. The patient ultimately obtained poor recovery of his left hearing loss (Figure 3C) (Table 3).

## 4. Discussion

This prospective cohort study attempted to unveil the association between EBV infection and sudden hearing loss. Our study demonstrated that approximately one-tenth of the enrolled SSNHL patients had evidence of detectable circulating EBV DNA, as reflected by the qPCR test results. The present study also demonstrated that there was a negative correlation between EBV DNA level and hearing recovery when investigating the EBV viral load and the hearing outcome of each PCR-positive individual. This is the first study in which PCR results were used to evaluate the correlation between idiopathic sudden hearing loss and EBV infection.

SSNHL is defined as sensorineural hearing impairment of 30 dB HL or more for at least three adjacent audiometric frequencies that develop within 72 h [28]. The incidence of SSNHL is approximately 5–20 per 100,000 people annually [29] and might be underestimated because of the affected individuals who recover without presenting to medical facilities [30,31]. Normal or complete recovery to functional hearing occurred in 45-65% of patients [30,32]. Identifiable etiologies are noted among 7% to 45% of individuals [3,29,30,32,33,34,35]; therefore, the majority of cases are idiopathic. Several studies have proposed etiologies for SSNHL that include vascular compromise, cochlear membrane rupture, and viral infection [29,36,37].

Different lines of evidence, including clinical studies, animal studies, and histopathological studies, have found viral infection or viral reactivation within the inner ear as the cause of cochlear inflammation or damage [29,38]. Elevated levels of serum antibodies to certain viruses, including herpes zoster, herpes simplex type 1 (HSV1), enterovirus, cytomegalovirus, rubeola, mumps, influenza B, and human papillomavirus (HPV), have been observed in idiopathic SSNHL patients [14,17,22,28,39,40,41,42]. EBV is one of the most common viruses that infects over 90% of the population worldwide and is especially prevalent in East Asia, including Taiwan [43,44]. The direct association between EBV infection and SSNHL remains controversial. Some case reports found a possible role for EBV infection [23,45,46]; however, Gross et al. [22] failed to support this finding. They found that for IgM antibodies against EBV-specific VCA from 48 unselected SSNHL patients, only 3 patients (6.2%) had positive results. In contrast to Gross et al., among the 29 SSNHL patients in our cohort study, 12 patients (41.4%) had detectable IgA titers against EBV-specific VCA (≥1:40), including 9 patients who exhibited a titer of 1:40, 2 patients who had a titer of 1:80, and 1 patient who had a titer of 1:160. On the other hand, one patient (3.4%) exhibited elevated IgA titers of 1:80 and 1:40 against both an EBV-specific EA and an EBV-specific VCA, respectively. Data regarding IgA antibodies in SSNHL patients with EBV are still lacking. However, viral variation and genetic susceptibility in different geographic areas might have a role in this result, from our perspective. Polz et al. [47] demonstrated that the prevalence of EBV genotypes differed among Taiwanese, Polish, and Arabic healthy individuals, according to which 62.5% of the Taiwanese and 55.6% of the Polish population had the same EBV LMP-1 gene variant; however, this same allele was not present in the Arab population.

A strong association between elevated IgA antibodies against EBV-specific VCA and EA and the risk of nasopharyngeal cancer (NPC) has been well demonstrated [48,49,50]. However, one of the primary obstacles to substantiating the association between viral infection and SSNHL may be the low sensitivity of immunoglobulin assays that previous studies have used. In a study by Scalia et al. [39], 31 of 93 SSNHL patients had IgA titers >1:80. This demonstrated that serum IgA antibody levels against HSV1-specific VCA that are higher than 1:80 are suggestive of an association of HSV1 infection with SSNHL. Our study revealed that nine patients (31%) had a titer of 1:40, two patients (6.9%) had a titer of 1:80, and only one patient (3.4%) had a titer of 1:160. Among the EB DNA-positive group (three patients), two (66.7%) showed undetectable VCA-IgA, and all three (100%) showed undetectable EA-IgA. The low titers and ratio of detectable IgA among the positive EB DNA PCR groups might be related to the natural kinetics of antibody formation occurring later than viral load elevation [51]. Thus, our study found the less diagnostic potential of IgA as a marker for EBV in SSNHL patients than for HSV1. Therefore, we obtained serum samples containing DNA from circulating EBV and tested them using PCR. This method has a high sensitivity for primary EBV infection detection [52,53,54,55] and is negative in post-infection cases [56,57,58]. Our study revealed that three patients (10.3%) had evidence of primary EBV infection when diagnosed with SSNHL. In a comparison study on NPC patients with healthy controls conducted in Taiwan by Lin et al., plasma EB DNA was not detectable in 40 healthy controls, with detectable DNA in 94 of the 99 advanced NPC patients [59]. Walton et al. reported that 0.6% of healthy controls (1 out of 165 people) had EBV DNA detected in plasma [60]. Kanarky et al. reported that in patients with active, systemic EBV(+) diseases (n = 105), EBV was detected in plasma in 99% of the cases [61]. In this regard, the high prevalence of detectable EBV DNA in SSNHL patients in our study (10.3%) supported, at least in part, the viral etiology of SSNHL, in addition to other popular theories such as vascular compromise and cochlear membrane rupture [29,36,37].

Our study showed that EB PCR-positive SSNHL cases might present an audiometric curve shape similar to that of EB PCR-negative cases. The initial severity and audiometric curve shape have been associated with prognosis in SSNHL. Ascending audiograms might have a better prognosis, and flat and profound audiograms have a poor prognosis, which was hypothesized to be due to the different susceptibilities of hair cells between the basal turn and apex [62]. Inconsistent with the literature, our three PCR-positive cases with flat and profound types showed different degrees of recovery. In our prospective study, the difference in viral burden was still a possible contributive factor, which may be explained by viral invasion or reactivation of latent virus within the ear. However, a lack of previous studies focused on the viral load, and the hearing recovery of SSNHL was noted. Scalia et al. [39] studied hearing outcomes based on HSV IgA titers at a cutoff value of 1:80, but different therapies were applied in the two groups. Acyclovir monotherapy was prescribed to HSV IgA > 1:80 patients. The inaccessibility of the cochlea in living patients was also a major obstacle to proving our hypothesis.

Our study revealed a negative trend between the EBV DNA level and hearing gain with systemic corticosteroid therapy, including one positive case with the highest serum level of EBV DNA that showed no improvement after steroid therapy. Our finding is consistent with a previous study indicating that EBV infection with a positive antibody test result should be considered a cause of SSNHL without any hearing recovery [24]. From our perspective, although systemic or intratympanic corticosteroids are recommended in cases of SSNHL suspected to have an inflammatory etiology [63,64], little is known about the steroid-responsive mechanisms in the ear, especially for the viral etiology. The protective effect of steroids in the inner ear was confirmed in animal models for acute acoustic trauma [65,66,67,68,69], certain drug cytotoxicity [70,71], pneumococcal meningitis [72], and autoimmune-associated hearing loss [73,74]. On the other hand, the effectiveness of systemic corticosteroids as monotherapy in EBV-associated infectious diseases, including infectious mononucleosis (IM) and hemophagocytic lymphohistiocytosis (HLH), has shown poor outcomes and limited benefit in recent studies [75,76]. Although our findings may be limited due to small patient numbers, it is rational that a high EBV serum level might be a poor prognostic factor for SSNHL patients with corticosteroid monotherapy, which also reflects the limitations of existing SSNHL studies to establish suitable treatments for different etiopathogeneses.

Given the effort to investigate the link between EBV and SSNHL, this study had several weaknesses. First, the small number of 29 patients, the lack of a healthy control group, and the absence of previous studies are weaknesses of the present study. Statistically significant differences may not have been detected due to the small number of participants. Having a healthy control group would inform us about the percentage of the healthy population with IgA antibodies and EB DNA. Further study with larger populations with serial serological test evaluations is needed to draw more definitive conclusions.

## 5. Conclusions

This is the first study to use real-time PCR to detect possible concurrent EBV infection in SSNHL populations in an East Asian population. Our study demonstrated that 10.3% of SSNHL patients showed detectable EBV DNA using PCR. Further prospective studies with larger populations and adequate control groups and hearing analyses among SSNHL patients with positive evidence of EBV infection are needed.

## Figures and Tables

**Table 1:** Baseline characteristics.

		Negative EBV PCR (n = 26)	Positive EBV PCR (n = 3)	*p*-Value
Age (mean, SD)		47 (14.9)	37 (14.7)	0.223
Sex (n, %)	Female	12 (46.2%)	2 (66.7%)	0.569
	Male	14 (53.8%)	1 (33.3%)	
IgA titer (n, %)	Undetectable	15 (57.7%)	2 (66.7%)	1.000
	Detectable	11 (42.3%)	1 (33.3%)	
VCA-IgA (n, %)	<1:40	15 (57.7%)	2 (66.7%)	
	1:40	9 (34.6%)	0	
	1:80	1 (3.8%)	1 (33.3%)	
	1:160	1 (3.8%)	0	
EA-IgA (n, %)	<1:40	25 (96.2%)	3 (100%)	
	1:80	1 (3.8%)	0	
Audiogram curve shape	Ascending	4 (15.4%)	0	
	Descending	2 (7.7%)	0	
	Flat	9 (34.6%)	1 (33.3%)	
	Profound	11 (42.3%)	2 (66.7%)	
Severity of hearing loss	Grade 1	1 (3.8%)	0	
	Grade 2	4 (15.4%)	0	
	Grade 3	8 (30.8%)	1 (33.3%)	
	Grade 4	4 (15.4%)	2 (66.7%)	
	Grade 5	9 (34.6%)	0	
Threshold at presentation (mean, SD)		72.7 (25.7)	80.0 (5.0)	0.813

**Table 2:** Hearing outcomes.

	Negative EBV PCR	Positive EBV PCR	*p*-Value
Complete recovery (CR)	8 (33.3%)	1 (33.3%)	
Partial recovery (PR)	2 (8.3%)	1 (33.3%)	
Slight improvement (SI)	3 (12.5%)	0	
No improvement (NI)	8 (33.3%)	1 (33.3%)	
Nonserviceable ear (NS)	3 (12.5%)	0	
Recovery threshold (mean, SD)	25.7 (20.1)	33.3 (32.9)	0.635

**Table 3:** Features of patients with positive PCR.

	Age	Sex	Severity	Audiogram Curve Shape	Threshold at Presentation (dB HL)	PCR Level (IU/mL)	Threshold Recovery after Treatment (dB)
Patient 1	39	F	Grade 3	Profound	80	35	61
Patient 2	26	F	Grade 3	Profound	85	59	42
Patient 3	46	M	Grade 3	Flat	75	473	−3

## Data Availability

The data presented in this study are available on request from the corresponding author. The data are not publicly available due to privacy.
